# Treatment of Paralysis

**Published:** 1840

**Authors:** 


*Treatment of Paralysis.*—A word in relation to the mode of treating paralysis, practised at this establishment. The spinal column is blistered from the neck to the sacrum, with the ordinary unguentum epispasticum, and the blistered surface is dressed with ungent. hydrag. Regimen according to the habit of each individual case. It is said that great success has attended the practice.—*Ib.*

